# Nipah Virus-Like Particle Egress Is Modulated by Cytoskeletal and Vesicular Trafficking Pathways: a Validated Particle Proteomics Analysis

**DOI:** 10.1128/mSystems.00194-19

**Published:** 2019-09-24

**Authors:** Gunner P. Johnston, Birgit Bradel-Tretheway, Paul D. Piehowski, Heather M. Brewer, Bom Nae Rin Lee, Nicholas T. Usher, J. Lizbeth Reyes Zamora, Victoria Ortega, Erik M. Contreras, Jeremy R. Teuton, Jason P. Wendler, Keesha M. Matz, Joshua N. Adkins, Hector C. Aguilar

**Affiliations:** a Department of Microbiology and Immunology, College of Veterinary Medicine, Cornell University, Ithaca, New York, USA; b Paul G. Allen Center for Global Animal Health, Washington State University, Pullman, Washington, USA; c Pacific Northwest National Laboratories, Richland, Washington, USA; Harvard Medical School

**Keywords:** Nipah virus, paramyxovirus, proteomics, vesicular trafficking, endocytosis, cytoskeleton, host-pathogen interaction

## Abstract

Classified as a biosafety level 4 (BSL4) select agent, Nipah virus (NiV) is a deadly henipavirus in the *Paramyxoviridae* family, with a nearly 75% mortality rate in humans, underscoring its global and animal health importance. Elucidating the process of viral particle production in host cells is imperative both for targeted drug design and viral particle-based vaccine development. However, little is understood concerning the functions of cellular machinery in paramyxoviral and henipaviral assembly and budding. Recent studies showed evidence for the involvement of multiple NiV proteins in viral particle formation, in contrast to the mechanisms understood for several paramyxoviruses as being reliant on the matrix (M) protein alone. Further, the levels and purposes of cellular factor incorporation into viral particles are largely unexplored for the paramyxoviruses. To better understand the involvement of cellular machinery and the major structural viral fusion (F), attachment (G), and matrix (M) proteins, we performed proteomics analyses on virus-like particles (VLPs) produced from several combinations of these NiV proteins. Our findings indicate that NiV VLPs incorporate vesicular trafficking and actin cytoskeletal factors. The involvement of these biological processes was validated by experiments indicating that the perturbation of key factors in these cellular processes substantially modulated viral particle formation. These effects were most impacted for NiV-F-modulated viral particle formation either autonomously or in combination with other NiV proteins, indicating that NiV-F budding relies heavily on these cellular processes. These findings indicate a significant involvement of the NiV fusion protein, vesicular trafficking, and actin cytoskeletal processes in efficient viral particle formation.

**IMPORTANCE** Nipah virus is a zoonotic biosafety level 4 agent with high mortality rates in humans. The genus to which Nipah virus belongs, Henipavirus, includes five officially recognized pathogens; however, over 20 species have been identified in multiple continents within the last several years. As there are still no vaccines or treatments for NiV infection, elucidating its process of viral particle production is imperative both for targeted drug design as well as for particle-based vaccine development. Developments in high-throughput technologies make proteomic analysis of isolated viral particles a highly insightful approach to understanding the life cycle of pathogens such as Nipah virus.

## Abstract (precis)

Nipah virus is a zoonotic biosafety level 4 agent with high mortality rates in humans. The genus to which Nipah virus belongs, Henipavirus, includes five officially recognized pathogens; however, over 20 species have been identified in multiple continents within the last several years. As there are still no vaccines or treatments for NiV infection, elucidating its process of viral particle production is imperative both for targeted drug design as well as for particle-based vaccine development. Developments in high-throughput technologies make proteomic analysis of isolated viral particles a highly insightful approach to understanding the life cycle of pathogens such as Nipah virus.

## INTRODUCTION

Nipah virus (NiV) is a pathogen in the family *Paramyxoviridae,* which includes measles and mumps viruses. It is highly virulent and capable of infecting numerous species of mammals. NiV particles are enveloped and transmissible to humans from fruit bat reservoirs as well as from livestock and other humans. Infection in humans leads to respiratory disease, severe encephalitis, and a case mortality rate between 40% and 100% (http://www.searo.who.int/entity/emerging_diseases/links/nipah_virus_outbreaks_sear/en/). Based on the absence of approved vaccines or treatments with the emerging threat that these viruses pose to human health, NiV and the closely related Hendra virus (HeV) are classified as biosafety level 4 (BSL4) agents and are included within the genus Henipavirus. Moreover, the World Health Organization recently listed NiV among the pathogens most likely to cause a major pandemic (1). Importantly, at least 20 new henipavirus-like viruses have been discovered in the last decade, underscoring the potential threat of NiV and related pathogens (2–4). The identification of pathogen-host interactions and elucidation of how these pathogens produce viral particles may yield novel antiviral therapeutics or support the development of particle-based vaccines such as those approved by the FDA for hepatitis B virus and human papillomavirus (5–7).

The formation of infectious paramyxovirus particles must include the incorporation of two kinds of transmembrane proteins, the fusion (F) and attachment (termed G for NiV and other henipaviruses) glycoproteins, which cooperate for viral entry in addition to the viral ribonucleoprotein complex, which includes the single-stranded, negative-sense viral genome and several viral proteins needed for transcription and replication (8). All paramyxoviruses additionally produce a matrix (M) protein which is generally thought to be central to the assembly and budding of viral components into new particles (9). Specifically, several paramyxovirus M proteins drive budding largely through oligomerization into a scaffolding array capable of inducing membrane curvature (10). While for some paramyxoviruses, the matrix protein is solely required to produce particles (11, 12), for Sendai virus, mumps virus, and simian virus 5, one or both glycoproteins are supportive of or essential for particle formation (13–16). Interestingly, live NiV containing a deletion of the M gene yielded a dramatic reduction in, but not complete loss of, infectivity (17). Further, potential roles for NiV proteins other than M in viral production have begun to be identified (18–21).

Unlike paramyxovirus matrix proteins, which appear to primarily use electrostatic interactions between M monomers to drive budding (10, 22, 23), other paramyxovirus proteins appear to instead reappropriate cellular machinery to accomplish budding (15, 19–21, 24). For example, proteins of the endosomal sorting complexes required for transport (ESCRTs) and associated factors are specifically targeted for recruitment to budding sites by numerous enveloped viruses which encode conserved motifs, termed late domains, within their viral proteins (19, 25–32). Generally, the removal or mutation of these late domains drastically reduces particle formation efficiency. Viral utilization of cellular factors other than ESCRTs for viral budding have also been reported, including Rab11 GTPase, involved in recycling endosome function, and known Rab11-interacting factors (33–35). Another cellular machinery known to be involved in several mechanisms of assembly and/or egress is the actin cytoskeleton; however, its roles vary greatly and, for many viruses, are poorly understood (24, 36–39), highlighting the many strategies viruses have adopted to ensure their replication and spread.

An approach to the identification and elucidation of roles for cellular machinery in enveloped particle formation is the use of proteomics to identify the host proteins enriched in viral particles and the cellular processes involved (40–42). A recent study by Vera-Velasco et al. focused on the identification of host cellular proteins in NiV virus-like particles (VLPs) produced from cotransfection of the NiV F, G, and M proteins (43). Among the insights gained in this study, 67 human proteins were identified that were primarily associated with vesicular transport and sorting. However, the importance of these cellular factors during viral particle formation and the involvement of each F, G, and M viral protein in the recruitment or incorporation of such cellular factors into VLPs remain elusive.

Based on two studies indicating roles of the NiV and HeV F proteins in particle formation (18, 44), we expanded this VLP proteomics approach to include several combinations of particles produced from the coexpression of F, G, and M. Our subsequent finding that F- but not M-derived VLPs incorporate numerous cellular factors adds support to the notion that henipaviral F proteins are involved in viral assembly and budding with a heavy reliance on cellular factors. Our results validate the importance of the most enriched processes, vesicular trafficking and the cytoskeleton, as modulatory of NiV particle release primarily through F-driven budding.

## RESULTS

### Overview of workflow and results from VLP and cellular proteomics.

**FIG 1:**
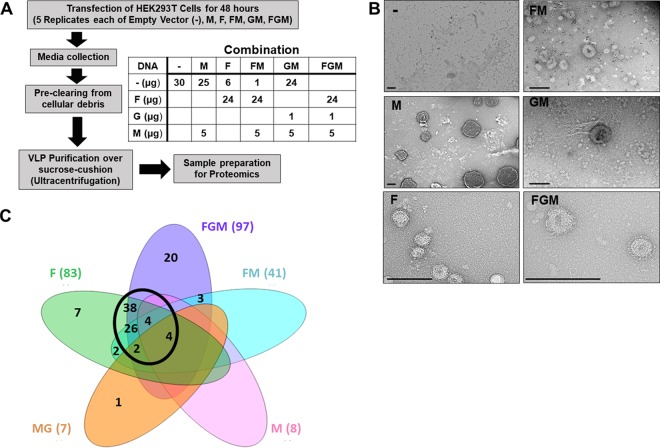
Overview of workflow and results from VLP and cellular omics. (A) Human embryonic kidney (HEK293T) cells were transfected with combinations of constructs coding for the Nipah virus fusion (F), attachment (G), and matrix (M) proteins. Media and cells were collected after 48 h. The medium supernatant was used to isolate virus-like particles (VLPs), and then both VLPs and cells were prepared for proteomics analysis. (B) VLPs produced from the listed combinations of viral protein transfection were isolated and negatively stained with 1.5% uranyl acetate. Scale bars = 250 nm. (C) Venn diagram summarizing the number of proteins identified as enriched in each VLP combination and their distribution of overlap. The black circle highlights the significant overlap between combinations including F.

To help identify specific cellular factors and machinery incorporated during NiV budding, we transfected human embryonic kidney (HEK293T) cells with plasmid constructs coding for individual structural NiV proteins, F, G, and M. To best elucidate the relative importance of the F and M proteins for incorporation of cellular factors, five combinations of transfections (F alone, M alone, F and M [FM], G and M [GM], and F, G, and M [FGM]) were completed in addition to an empty vector control, a background for naturally expressed extracellular vesicles (e.g., exosomes and microvesicles) (Fig. 1A). Forty-eight hours after transfection, VLPs and their corresponding transfected cells were separately isolated and prepared for proteomic analyses (Fig. 1A). As described in Materials and Methods, the observed peptide counts were used to remove replicates indicating contamination from overflow, resulting in 3 to 5 biological replicates per transfection type being used for further analyses. Average peptide counts were used to identify which cellular factors were incorporated into VLPs using two thresholds, a minimum average peptide count of 4.33 for the replicates of a given transfection type (e.g., F only), based on the average observed peptide counts of the viral proteins themselves, as well as a maximum average count of 2 in empty vector controls to remove proteins highly expressed in background extracellular vesicles. To verify that this approach produces vesicles and to visualize vesicles for each combination, negative staining and transmission electron microscopy were used (Fig. 1B). Importantly, spikes were most apparent in combinations containing F and/or G, supporting the incorporation of these proteins, whereas M-only VLPs lacked spikes and tended to include high-contrast structures internally (Fig. 1B). From the VLP proteomics, the following numbers of cellular proteins were identified for each VLP sample type: 8 (M), 83 (F), 41 (FM), 7 (MG), and 97 (FGM) (Fig. 1C). A Venn diagram was constructed to help identify cellular proteins that were common to all, some, or only one VLP combination.

### VLP proteomics highlighted by enrichment of cytoskeletal and endosomal trafficking machinery.

**FIG 2:**
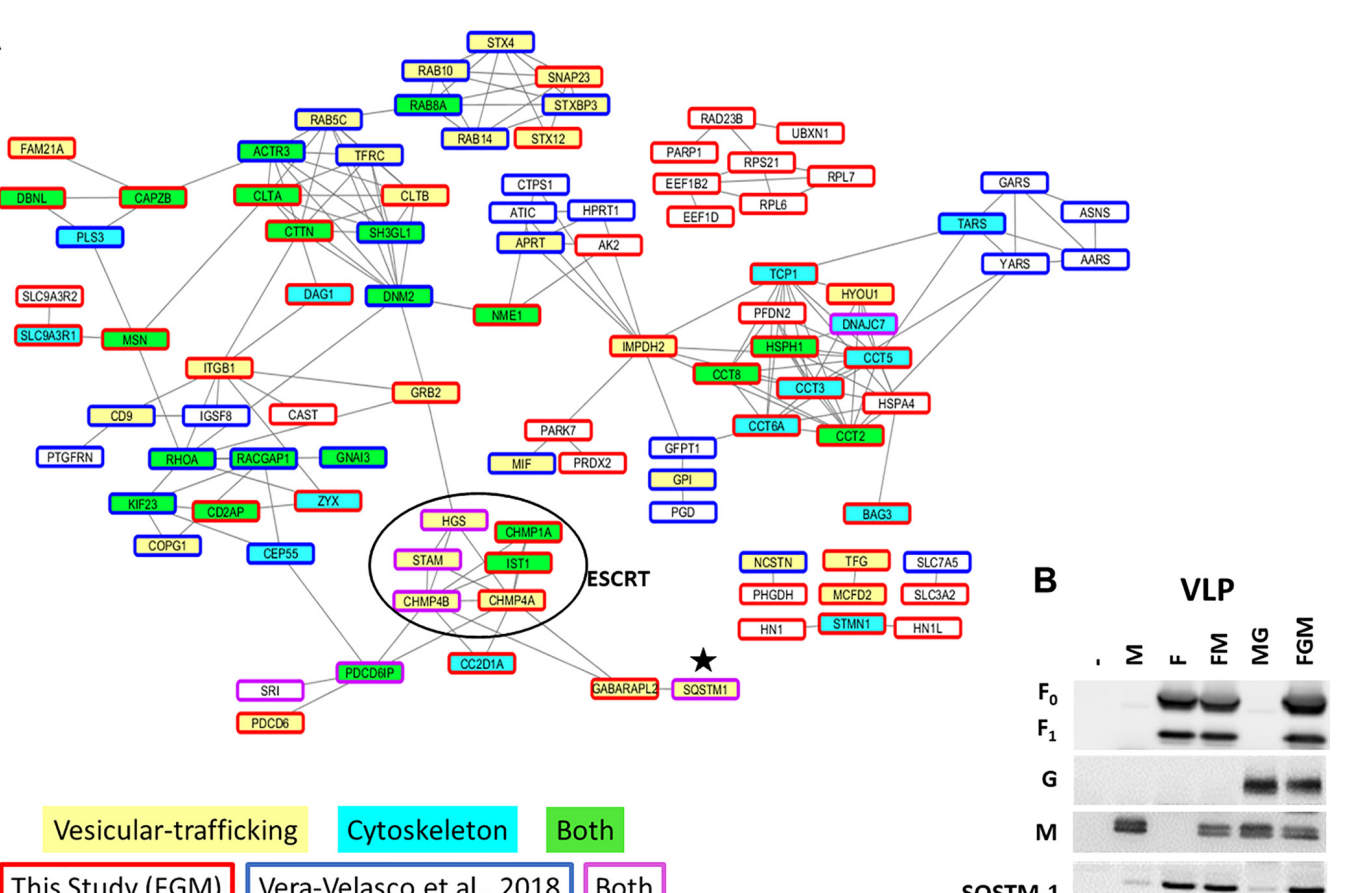
VLP proteomics highlighted by enrichment of cytoskeletal and endosomal trafficking machinery. (A) STRING protein interaction map comparing proteins identified in the FGM VLPs described by Vera-Velasco et al. (43) (blue box) and this study (red boxes). This map identifies which protein clusters have associated factors included in one or both (purple boxes) studies. Among the most enriched groups based on gene ontological analyses of the combined data set are vesicular trafficking (yellow) and the cytoskeleton (light blue), with proteins that belong to both also designated (green). ESCRTs which somewhat overlap both processes were observed in both studies and are in a black circle. Note that the clusters associated with these processes tend to have included proteins identified in both studies analyzed. Importantly, only proteins with at least one interaction were shown on this map; a full list can be found in Table 1. One of the most clearly enriched proteins in VLP proteomics for this study and that by Vera-Velasco et al. (43) was the autophagy adapter protein, sequestosome-1 (SQSTM1, designated by small black star). (B) Using Western blot analysis of VLPs for each combination, sequestosome-1 was used to validate the accuracy of our VLP proteomics, since this protein exhibited an expression pattern similar to the mass spectrometry results. The Western blot is representative of at least three replicates.

We next created a protein interaction map by combining our FGM VLP proteomics data with the data from a recent proteomics composition study on NiV FGM VLPs (43) to help us identify protein clusters consistently incorporated into VLPs. Overall, the enriched processes and protein-protein interaction sets have a high degree of overlap; however, specific proteins identified in common are relatively few. Small differences in specific protein compositions for VLPs are likely influenced by differences in methodologies; however, the consistent enrichment of several processes suggests their underlying involvement. Namely, two of the most enriched functional groups of proteins were vesicle-mediated transport and the actin cytoskeleton (Fig. 2A). Sequestosome-1 (SQSTM1), an adapter protein involved in vesicle-mediated transport, namely, macroautophagy (45), was found to be among the most enriched proteins, particularly in VLPs produced from cells expressing F. To support the validity of our VLP proteomics platform, the VLP samples used in proteomics were also analyzed by Western blotting, and the pattern of SQSTM1 incorporation primarily into VLPs containing NiV-F was confirmed (Fig. 2B). A full list of VLP-incorporated proteins is shown in Table 1, comparing the proteins identified in different types of VLPs (F, M, etc.) with those in FGM VLPs in a study by Vera-Velasco et al. (43) and further identifying whether each protein is associated with vesicle-mediated transport or the cytoskeleton. Comparisons between the lists of cellular proteins incorporated into each of the VLP types (Table 1) was primarily done to elucidate how much the budding mechanisms of F and M rely on cellular machinery.

**TABLE 1:** Genes and number of VLP incorporated proteins

Gene	Full name	Accession no.	Avg. no. of observed peptides for^*a*^:								
			VMT (65)	Cytoskeleton (54)	Negative control	M (8)	F (83)	FM (41)	MG (7)	FGM (97)	Vera-Velasco et al. (67)
AARS	Alanyl-tRNA synthetase	P49588									
ABCE1	ATP binding cassette subfamily E member 1	P61221									
ACTR3	ARP3 actin-related protein 3 homolog	F8WEW2									
AK2	Adenylate kinase 2	F8VZG5			0.8	0.7	6.0	4.3	1.4	5.5	
ALCAM	Activated leukocyte cell adhesion molecule	F5GXJ9			0.3	1.0	7.3	3.0	1.8	4.3	
AMOT	Angiomotin	A2BDD9			0.3	0.0	6.0	1.3	0.4	9.0	
ANXA6	Annexin A6	E5RFF0									
APRT	Adenine phosphoribosyltransferase	H3BQB1									
ASNS	Asparagine synthetase	C9J605									
ATIC	5-Aminoimidazole-4-carboxamide ribonucleotide formyltransferase/IMP cyclohydrolase	V9HWH7									
BAG3	BCL2-associated athanogene 3	O95817			0.0	0.0	19.3	9.7	0.2	23.3	
CA2	Carbonic anhydrase 2	V9HW21			0.3	2.7	2.5	3.3	3.8	5.5	
CALCOCO2	Calcium binding and coiled-coil domain 2	H0YBP4			0.0	0.0	10.0	4.7	0.8	10.3	
CALM1	Calmodulin 1	Q96HY3			2.0	4.0	5.0	4.3	4.0	4.3	
CALU	Calumenin	A0A024R755			0.0	0.0	8.0	3.3	2.0	8.3	
CAPZA1	Capping actin protein of muscle Z-line alpha subunit 1	P52907			0.5	0.7	4.5	2.3	1.2	4.3	
CAPZB	Capping actin protein of muscle Z-line beta subunit	B1AK87			2.0	1.7	6.3	3.3	2.4	5.0	
CAST	Calpastatin	E7ES10			0.0	0.0	3.5	1.0	0.4	5.0	
CBR1	Carbonyl reductase 1	E9PQ63									
cc2d1a	Coiled-coil and C2 domain containing 1A	K7EJY5			0.0	0.0	5.5	3.3	0.2	7.5	
CCT2	Chaperonin containing TCP1 subunit 2	F8VQ14			0.5	1.3	4.8	4.3	2.2	6.3	
CCT3	Chaperonin containing TCP1 subunit 3	P49368			1.3	2.0	5.8	4.3	2.6	5.5	
CCT5	Chaperonin containing TCP1 subunit 5	E7ENZ3			1.5	3.3	6.3	5.7	3.2	7.3	
CCT6A	Chaperonin containing TCP1 subunit 6A	P40227			0.8	0.3	4.3	4.7	1.8	5.5	
CCT8	Chaperonin containing TCP1 subunit 8	H7C2U0			1.5	0.7	5.0	4.3	1.8	7.5	
CD276	CD276 molecule	H0YN85									
CD2AP	CD2-associated protein	Q9Y5K6			0.0	1.3	8.0	3.3	1.0	10.5	
CD9	CD9 molecule	B4DK09									
CDC37	Cell division cycle 37	Q16543			0.0	1.0	2.3	2.7	0.2	5.0	
CDV3	CDV3 homolog	D6R973			0.0	1.0	6.3	4.3	1.8	7.5	
CEP55	Centrosomal protein 55	Q53EZ4									
CHMP1A	Charged multivesicular body protein 1A	F8VUA2			0.0	0.0	4.0	2.7	0.6	4.5	
CHMP4A	Charged multivesicular body protein 4A	Q9BY43			0.3	1.0	5.3	3.0	1.2	6.3	
CHMP4B	Charged multivesicular body protein 4B	Q9H444			1.3	2.3	10.0	7.3	3.2	10.5	
CLIC4	Chloride intracellular channel 4	Q9Y696									
CLTA	Clathrin light chain A	C9J8P9			0.8	2.7	5.8	3.7	4.2	6.3	
CLTB	Clathrin light chain B	D6RJD1			0.0	0.7	3.0	1.7	0.4	5.0	
COPG1	Coatomer protein complex subunit gamma 1	D6RG17									
CPNE1	Copine-1	Q99829									
CPNE3	Copine-3	E5RFT7									
CTPS1	CTP synthase 1	P17812									
CTTN	Cortactin	H0YCD9			0.3	3.7	13.3	6.7	4.2	14.5	
CXADR	Coxsackievirus and adenovirus receptor	P78310			0.3	2.0	5.5	3.3	1.2	5.5	
DAG1	Dystroglycan 1	C9JQL4			0.0	1.7	5.5	2.7	0.6	8.3	
DBI	Diazepam binding inhibitor, acyl-CoA binding protein^*b*^	B8ZWD8			1.8	6.0	7.8	4.3	2.6	5.0	
DBN1	Drebrin 1	D6RCR4			0.3	1.3	3.8	1.0	2.0	4.5	
DBNL	Drebrin-like	H7C111			0.0	0.7	4.8	1.7	1.0	4.5	
DNAJC7	DnaJ heat shock protein family	K7EQ73			0.0	0.0	5.0	2.7	0.0	6.0	
DNM2	Dynamin 2	K7EMR9									
DSG2	Desmoglein 2	J3KSI6			1.3	5.3	16.5	10.0	4.8	18.5	
DYNC1LI1	Dynein cytoplasmic 1 light-intermediate-chain 1	Q9Y6G9									
EEF1B2	Eukaryotic translation elongation factor 1 beta 2	F8WFC9			1.5	5.3	8.3	6.3	5.6	7.8	
EEF1D	Eukaryotic translation elongation factor 1 delta	Q9BW34			1.8	4.0	10.3	5.7	3.4	9.8	
EHD4	EH domain containing 4	Q9H223									
EPB41	Erythrocyte membrane protein band 4.1	Q4VB87									
EPB41L2	Erythrocyte membrane protein band 4.1-like 2	E9PMG5			0.8	1.3	3.3	1.7	3.0	5.5	
ERP29	Endoplasmic reticulum protein 29	F8W1G0			0.5	1.7	7.8	4.3	0.6	7.5	
fam21a	Family with sequence similarity 21 member A	Q641Q2			0.0	0.0	6.8	2.0	1.8	8.3	
FLOT2	Flotillin 2	Q14254									
GABARAPL2	GABA type A receptor-associated protein-like 2	H3BU36			0.0	0.0	6.0	3.0	0.2	5.3	
GARS	Glycyl-tRNA synthetase	A0A090N8G0									
GFPT1	Glutamine–fructose-6-phosphate transaminase 1	Q06210									
GIPC1	GIPC PDZ domain containing family member 1	K7EJ33			0.0	1.0	3.5	1.7	1.2	5.5	
gnai3	G protein subunit alpha i3	P08754									
GNAS	GNAS complex locus	Q5JWF2									
GOLM1	Golgi membrane protein 1	C9J941			0.5	1.3	5.3	3.0	2.0	5.3	
GPI	Glucose-6-phosphate isomerase	A0A0J9YXP8									
GRB2	Growth factor receptor-bound protein 2	J3QRL5			0.5	0.0	5.5	2.0	0.8	5.8	
HGS	Hepatocyte growth factor-regulated tyrosine kinase substrate	I3L1E3			0.0	0.3	4.3	2.0	2.8	5.8	
HINT1	Histidine triad nucleotide binding protein 1	H0YC49			0.0	2.3	4.5	2.7	2.0	4.0	
HN1	hematological and neurological expressed 1	J3KT51			0.8	2.3	4.8	3.0	2.4	4.5	
HN1L	Hematological and neurological expressed 1-like	Q9H910			0.3	1.7	4.5	4.0	1.6	5.3	
HPRT1	Hypoxanthine phosphoribosyltransferase 1	P00492									
HSP90AB3P	Heat shock protein 90 alpha family class B member 3, pseudogene	Q58FF7			1.0	1.0	4.8	3.3	2.2	5.3	
HSPA4	Heat shock protein family A	P34932			1.0	3.0	4.5	2.3	1.6	4.8	
HSPH1	heat shock protein family H	A0A024RDS1			0.8	1.3	7.5	4.3	1.6	8.3	
HYOU1	Hypoxia upregulated 1	A0A087WW13			1.3	1.3	4.3	3.0	1.0	5.8	
IGSF8	Immunoglobulin superfamily member 8	Q969P0									
IMPDH2	Inosine monophosphate dehydrogenase 2	Q6QE17			0.3	2.3	8.8	5.0	2.8	6.3	
IPO5	Importin 5	C9J875									
IST1	IST1, ESCRT-III-associated factor	J3QQP8			0.3	2.3	8.5	3.3	1.4	8.0	
ITGB1	Integrin subunit beta 1	P05556			1.3	2.3	4.3	4.3	2.4	6.8	
KHSRP	KH-type splicing regulatory protein	M0QYH3			1.3	2.7	4.3	4.3	1.4	7.8	
KIF23	Kinesin family member 23	H7BYN4									
LMNB1	Lamin B1	B4DZT3			0.5	2.0	2.3	4.0	0.8	5.8	
MAPRE1	Microtubule-associated protein RP/EB family member 1	A2VCR0			0.0	0.3	6.0	3.0	1.6	4.0	
MARCKSL1	MARCKS-like 1	P49006									
MCFD2	Multiple coagulation factor deficiency 2	H7BZ18			0.3	3.3	7.8	5.0	2.6	5.8	
MIF	Macrophage migration inhibitory factor	I4AY87									
MSN	Moesin	V9HWC0			0.5	4.0	5.3	5.3	3.6	6.3	
NASP	Nuclear autoantigenic sperm protein	E9PKR5			0.0	0.0	2.8	1.0	1.8	5.0	
NCSTN	Nicastrin	Q5T205			0.3	1.3	2.8	2.3	1.0	2.8	
NDRG1	*N*-*myc* downstream-regulated 1	Q8N959			0.0	0.3	6.3	3.3	1.2	7.5	
NENF	Neudesin neurotrophic factor	Q9UMX5			0.3	1.0	4.5	2.0	0.4	6.0	
NME1	NME/NM23 nucleoside diphosphate kinase 1	E7ERL0			0.5	2.3	7.0	3.3	2.2	5.5	
NSFL1C	NSFL1 cofactor	R4GNE6			0.0	0.7	6.5	1.3	0.4	6.8	
NUCB2	Nucleobindin 2	E9PLR0			1.5	3.7	9.8	7.0	3.0	10.3	
OLA1	Obg-like ATPase 1	J3KQ32									
PARK7	Parkinsonism-associated deglycase	K7EN27			0.0	2.7	8.0	5.0	2.6	7.5	
PARP1	Poly (ADP-ribose) polymerase 1	Q5VX85			0.5	4.7	5.8	7.3	2.8	11.0	
PDCD6	Programmed cell death 6	A0A024QZ42			0.8	1.7	5.5	5.3	1.4	4.5	
PDCD6IP	Programmed cell death 6-interacting protein	F8WBR8			1.5	2.3	10.3	11.7	4.4	11.8	
PFDN2	Prefoldin subunit 2	Q9UHV9			0.0	1.0	4.5	2.7	1.8	5.0	
PGD	Phosphogluconate dehydrogenase	K7EMN2									
PHGDH	Phosphoglycerate dehydrogenase	Q5SZU1			0.8	1.0	4.3	3.7	1.4	5.0	
PLD3	Phospholipase D family member 3	Q8IV08									
PLS3	Plastin 3	H7C4N2									
PPA1	Pyrophosphatase	Q5SQT6			0.0	0.0	4.0	1.7	1.4	4.8	
PPIB	Peptidylprolyl isomerase B	P23284			1.0	2.3	6.3	2.7	1.0	5.0	
PRDX2	Peroxiredoxin 2	A0A024R7F2			0.0	1.0	9.5	3.3	1.6	6.0	
PRDX4	Peroxiredoxin 4	A6NG45			1.8	5.7	10.5	7.0	1.6	7.8	
PRKCSH	Protein kinase C substrate 80K-H	A0A0S2Z4D8			0.0	0.7	6.8	2.3	0.6	5.5	
PSMB6	Proteasome subunit beta 6	Q6IAT9									
PTGFRN	Prostaglandin F2 receptor inhibitor	A4QPA1									
RAB10	RAB10, member of RAS oncogene family	Q9UL28									
RAB14	RAB14, member of RAS oncogene family	P61106									
RAB5C	RAB5C, member of RAS oncogene family	F8VVK3									
RAB8A	RAB8A, member of RAS oncogene family	P61006									
RACGAP1	Rac GTPase-activating protein 1	F8VZ66									
RAD23B	RAD23 homolog B, nucleotide excision repair protein	Q5W0S5			0.8	5.0	12.5	6.0	3.2	10.3	
RCN1	Reticulocalbin 1	E9PLM2			0.0	0.0	3.8	2.0	1.6	5.3	
RCN2	Reticulocalbin 2	L8E7F0			0.0	0.0	6.0	2.3	2.0	4.5	
RHOA	*ras* homolog family member A	C9JX21									
RPL15	Ribosomal protein L15	E7ERA2			2.0	3.7	6.0	3.3	3.2	3.3	
RPL6	Ribosomal protein L6	F8VU16			1.8	3.3	8.0	7.7	5.6	9.5	
RPL7	Ribosomal protein L7	P18124			1.5	2.0	5.5	5.3	3.0	6.3	
RPS18	Ribosomal protein S18	J3JS69			1.3	3.7	5.0	4.3	3.6	3.3	
RPS21	Ribosomal protein S21	Q6FGH5			0.5	2.0	4.3	2.7	1.4	5.0	
SDCBP	Syndecan binding protein	E9PBU7									
SDF4	Stromal cell-derived factor 4	Q9BRK5			0.0	1.3	6.5	4.0	2.2	7.8	
SH3GL1	SH3 domain containing GRB2-like 1, endophilin A2	Q9BVL7									
SLC12A2	Solute carrier family 12 member 2	B7ZM24			1.5	2.3	4.8	1.0	1.2	5.0	
SLC16A1	Solute carrier family 16 member 1	A0A024R0H1									
SLC39A14	Solute carrier family 39 member 14	A0A0S2Z5C8									
SLC3A2	Solute carrier family 3 member 2	F5GZS6			1.0	3.3	6.5	4.0	3.8	8.8	
SLC7A5	Solute carrier family 7 member 5	Q01650									
SLC9A3R1	SLC9A3 regulator 1	O14745			0.8	5.0	15.0	5.0	5.2	12.0	
slc9a3r2	SLC9A3 regulator 2	Q15599			0.0	0.0	1.8	0.3	0.8	6.0	
SNAP23	Synaptosome-associated protein 23	O00161			0.3	2.0	13.0	7.0	3.2	12.5	
SQSTM1	Sequestosome 1	E7EMC7			0.0	1.7	12.8	5.0	1.2	10.5	
SRI	Sorcin	C9J0K6			0.3	1.3	8.0	5.0	2.4	7.8	
STAM	Signal-transducing adapter molecule	B4DZT2			0.0	1.3	3.8	2.3	2.0	5.0	
STMN1	Stathmin 1	A2A2D0			1.3	8.0	8.5	8.0	4.4	12.0	
STX12	Syntaxin 12	Q86Y82			0.0	0.7	6.8	4.0	0.6	7.3	
STX4	Syntaxin 4	Q12846									
STXBP3	Syntaxin binding protein 3	O00186									
TARS	Threonyl-tRNA synthetase	D6RCS6									
TAX1BP1	Tax1 binding protein 1	C9JBZ7			0.0	0.0	8.5	3.0	0.8	7.0	
TBCA	Tubulin folding cofactor A	O75347			0.5	2.0	3.8	2.7	3.6	6.5	
TCP1	T-complex 1	F5H7Y1			0.8	0.3	4.8	4.3	2.4	6.8	
TF	Transferrin	C9JB55			1.5	1.3	1.8	2.7	4.8	2.0	
TFG	TRK-fused gene	Q92734			0.0	4.0	12.8	7.7	4.2	12.5	
TFRC	Transferrin receptor	A8K6Q8									
TOLLIP	Toll-interacting protein	Q6FIE9			0.3	1.0	5.3	3.7	1.2	3.5	
TOM1	Target of Myb1 membrane trafficking protein	H7BYN7			0.0	0.3	6.0	4.7	1.6	6.3	
TPD52L2	Tumor protein D52-like 2	O43399			0.0	1.7	5.3	2.3	1.8	4.5	
TSPAN3	Tetraspanin 3	L8E893									
TSPAN6	Tetraspanin 6	A0A024RCI0									
TXNDC12	Thioredoxin domain containing 12	V9GY50			0.5	0.7	5.5	3.0	0.6	4.5	
UBXN1	UBX domain protein 1	Q04323			0.0	0.7	5.0	1.3	0.8	5.0	
UCHL1	Ubiquitin C-terminal hydrolase L1	P09936			0.0	4.0	8.5	3.7	3.2	6.8	
USP5	Ubiquitin-specific peptidase 5	A0A140VJZ1									
VPS35	VPS35, retromer complex component	Q96QK1									
VTA1	Vesicle trafficking 1	Q9NP79			0.0	0.0	5.8	1.7	1.0	2.5	
YARS	Tyrosyl-tRNA synthetase	A0A0S2Z4R1									
ZYX	Zyxin	H7C3R3			0.0	1.0	3.8	3.7	2.4	8.5	

a Proteins identified in VLPs from at least one type of transfection and/or Vera-Velasco et al. (43) are listed along with their UniProt identifier, totaling 166 proteins. The inclusion of any given protein in any of these sample sets or in the Gene Ontology (GO) groups of vesicle-mediated transport (VMT) or cytoskeleton is indicated by gray shading. White shading indicates that the protein of interest was not identified in that group. The number of proteins identified in each transfection or GO set from the full list of 166 proteins is indicated in the header for the transfection or GO group.

b CoA, coenzyme A; EB, elementary body; RP, replicative phase; GABA, gamma-aminobutyric acid.

### Nipah virus F-driven budding is reduced upon inhibition of endocytic machinery.

**FIG 3:**
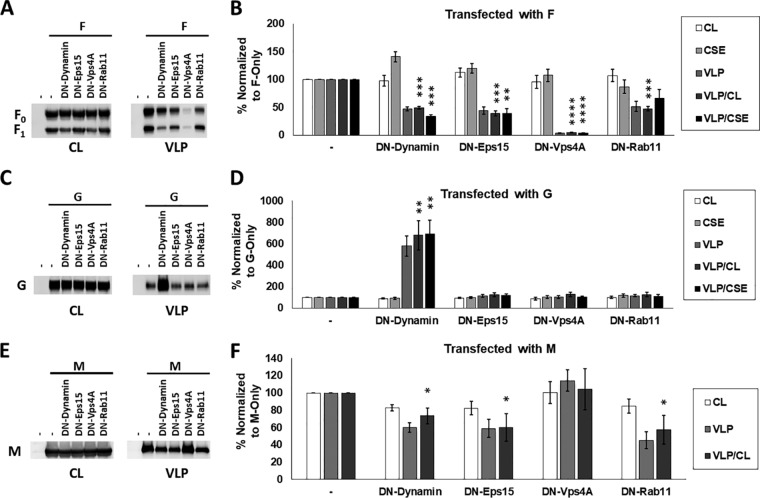
Nipah virus budding is significantly modulated upon the inhibition of endocytosis, recycling, and ESCRT function. (A) HEK293T cells were transfected with NiV F alone or with dominant-negative (DN) mutants of the following key vesicular trafficking factors: dynamin (endocytosis), Eps15 (clathrin-mediated endocytosis), VPS4A (ESCRT function), or Rab11 (recycling). Total cell lysates (CL) and virus-like particle (VLP) fractions were prepared 24 h after transfection and used for SDS-PAGE and Western blot analysis, whereas F cell surface expression (CSE) was measured using flow cytometry. (B) Using densitometry to assess the expression of the F protein in VLPs along with corresponding total cell lysate and CSE values, two indices were determined, VLP/CL and VLP/CSE. (C and D) As with panels A and B, a construct encoding G was coexpressed with each DN factor and the following CL, CSE, VLP, and index values elucidated. Similarly, coexpression with these constructs were tested for effects on M expression in cell lysates and VLPs but not at the cell surface due to it being a cytoplasmic protein. (E and F) Representative Western blots (E) and quantification (F) are shown. The results are representative of at least three experiments, with error bars indicating the standard error of the mean. One-way Student *t* tests were used to assess significant differences in budding efficiency indices compared to when they are cotransfected with pcDNA3.1, an empty vector (*, *P* < 0.05; **, *P* < 0.01; ***, *P* < 0.001; ****, *P* < 0.0001).

Based on our proteomics analyses, F seemed more likely than M to utilize cellular machinery for its budding function. To further validate proteomics and determine whether NiV F-driven budding is dependent on machinery involved in vesicular trafficking, we measured F expression in cell lysates (CL), at the cell surface (CSE), and in viral particles (VLP) after cotransfection with an empty vector (positive control, set to 100%) or the following dominant-negative (DN) constructs to inhibit several pathways of vesicular trafficking: DN-dynamin (various endocytic pathways), DN-Eps15 (clathrin-mediated endocytosis), DN-Vps4A (function of ESCRT complexes involved in multivesicular body formation and several mechanisms of viral budding), and DN-Rab11 (primarily recycling from endosomal system to the plasma membrane) (Fig. 3A). After quantification of these parameters with flow cytometry or densitometry, two budding indices, one based on each measurement of cellular expression, were calculated to assess the actual budding efficiencies under each transfection condition, VLP/CL and VLP/CSE, with the positive control set to 100% for each budding index.

We report that F budding VLP/CL and/or VLP/CSE efficiencies were significantly reduced to a range of 4 to 49% compared to the positive controls (assessed with one-sample *t* tests) after coexpression with each of these constructs (Fig. 3B). The only index not meeting significance was VLP/CSE for DN-Rab11, which still appeared to trend toward a reduction (reduced to 66%, *P = *0.13), with the VLP/CL reduction for DN-Rab11 being to 52.67%, *P = *0.0009). F budding was most affected by DN-Vps4A, indicating the importance of ESCRT function in its budding mechanism, whether directly or indirectly. Since this study additionally focused on how these factors might affect G and M budding efficiencies, they were similarly tested except that CSE cannot be measured for NiV M, which is not a transmembrane protein. Of significant interest, NiV G budding efficiency increased almost 7-fold based on both indices in the case of cotransfection with DN-dynamin but did not change under any other condition (Fig. 3C and D). This result was entirely unexpected and suggests a novel function of dynamin, direct or indirect, in the unelucidated and normally relatively inefficient process of G-driven particle formation (Fig. 3C) (18, 46). Compared to F and G, the effects of these constructs on M budding were less dramatic, though DN-dynamin, DN-Eps15, and DN-Rab11 coexpression led to significant (*P < *0.05) M budding efficiency reductions by 27%, 40%, and 42%, respectively (Fig. 3E and F). Importantly, the finding that DN-Vps4a does not affect NiV M budding efficiency is consistent with prior literature (47).

### Nipah virus budding is significantly affected by actin cytoskeletal manipulation through mutant RhoGTPases.

**FIG 4:**
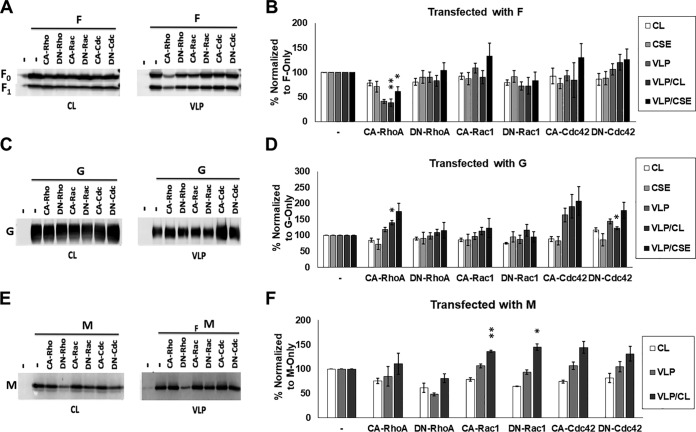
Nipah virus budding is significantly affected by actin cytoskeletal manipulation through mutant RhoGTPases. (A and B) HEK293T cells were transfected with NiV F alone or with dominant-negative (DN) or constitutively active (CA) constructs for RhoA, Rac1, and Cdc42. As with Fig. 3, CL, CSE, and VLPs were quantified and both budding indices assessed. SDS-PAGE and Western blot analysis. (C to F) The effects of coexpressing these mutants were assessed for G-driven (C and D) and M-driven (E and F) budding. The results are representative of at least three experiments, with error bars indicating the standard error of the mean. One-way Student *t* tests were used to assess significant differences in budding efficiency indices compared to when they are cotransfected with pcDNA3.1, an empty vector (*, *P* < 0.05; **, *P* < 0.01).

Actin cytoskeletal factor enrichment was largely specific to F alone and FGM VLPs in our proteomics analysis, which suggests that these factors may be recruited specifically by the presence of F to sites of assembly and budding. The actin cytoskeleton is involved in many cellular processes (48, 49), as well as assembly and/or egress for some viruses; however, these roles vary greatly and, for many viruses, are poorly understood (24, 36–39). The actin cytoskeleton is composed of monomeric globular (G-actin) and polymeric filamentous actin (F-actin), which are interconverted and organized by remodeling proteins, including RhoGTPases, to form specific microdomains within a cell (50–53). Major reorganization of the actin cytoskeleton into structures such as stress fibers, lamellipodia, and filopodia is regulated by the RhoGTPases RhoA, Rac1, and Cdc42, respectively (40). For an initial test of whether NiV F budding is modulated by the activity of RhoGTPases, we assessed whether F budding efficiency indices (VLP/CL and VLP/CSE) are altered during the coexpression of constitutively active (CA) and dominant-negative (DN) mutants of RhoA, Rac1, or Cdc42. We found that CA-RhoA reduced the levels of F in VLPs (VLP/CL to 54% [*P < *0.01] and VLP/CSE to 61% [*P < *0.05]) (Fig. 4A and B). We also tested the effects of these constructs on G- and M-driven budding. The G VLP/CL budding indexes following CA-RhoA and DN-Cdc42 coexpression were 140% and 122%, respectively (Fig. 4C and D). While the significance of inefficient G-driven budding is not understood, these findings may yield some insight into the processes involved. Interestingly, M budding was found to be increased by both CA-Rac1 and DN-Rac1, suggesting that perturbation of the function of this GTPase may support particle formation (Fig. 4E and F).

### Inhibition of Nipah virus fusion protein budding reduces attachment protein incorporation regardless of matrix protein expression.

**FIG 5:**
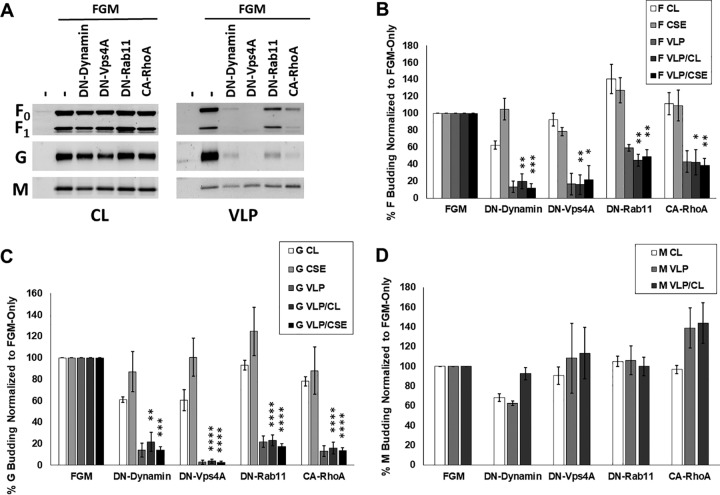
Inhibition of Nipah virus fusion protein budding reduces attachment protein incorporation despite the presence of normally budding matrix protein. (A) To assess the effects that manipulation of vesicular trafficking (Fig. 3) and the actin cytoskeleton (Fig. 4) have on more complete budding particles, NiV F, G, and M were cotransfected with or without DN-dynamin, DN-Vps4a, DN-Rab11, or CA-RhoA. The expression of each viral protein in cell lysates, on the cell surface for F and G, and in VLPs was quantified and used to produce budding indices as done previously. (B to D) These values are summarized for F (B), G (C), and M (D). The results are representative of at least three experiments, with error bars indicating the standard error of the mean. One-way Student *t* tests were used to assess significant differences in budding efficiencies for each viral protein compared to FGM coexpression alone (*, *P* < 0.05; **, *P* < 0.01; ***, *P* < 0.001; ****, *P* < 0.0001).

**FIG 6:**
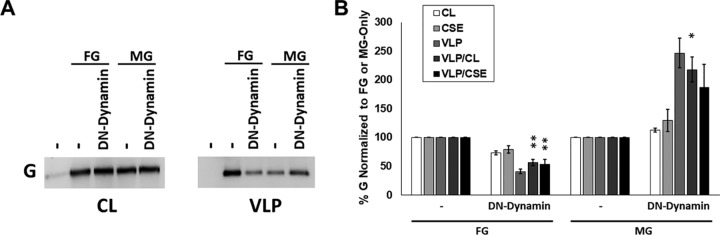
Loss of increased G incorporation from DN-dynamin expression occurs with expression of F but not G. HEK293T cells were transfected with constructs for NiV G and either F or M with or without the additional expression of DN-dynamin. As with Fig. 3 and 5, VLPs were collected compared with total and surface expression to assess the G budding efficiency. (A and B) Western blots (A) along with densitometric and surface expression (B) were quantified. The results are representative of at least three experiments, with error bars indicating the standard error of the mean. One-way Student *t* tests were used to assess significant differences in budding efficiencies for each viral protein compared to FGM coexpression alone (*, *P* < 0.05; **, *P* < 0.01).

To better elucidate the importance of the mutant factors tested so far, several of the most interesting factors were also assessed in the context of F, G, and M coexpression, which should better emulate particles produced in live NiV infections (Fig. 5A). While the FGM particles produced from this combination lack the internal viral genome ribonucleoprotein (vRNP) complex responsible for infection, they would contain all viral machinery needed for both entry (both F and G) and incorporation (M) of the vRNP. Despite the presence of the M protein, generally considered to be the most important viral protein in the process of paramyxovirus budding, we found that DN-dynamin, DN-Vps4a, DN-Rab11, and CA-RhoA all significantly and consistently reduced both indices of F budding efficiency by 51% to 88% (Fig. 5A and B). More strikingly still, we report that these reductions were also seen for NiV G in the context of F and M expression (FGM samples), which exhibited no less than a 77% reduction in budding efficiency in any tested combination (Fig. 5A and C). This was particularly interesting since only DN-dynamin and CA-RhoA appeared to previously affect G-driven budding, and both led to increased budding efficiency. M budding efficiencies, on the other hand, were not significantly affected by any coexpressed cellular factor while in this combination (Fig. 5A and D). Importantly, these findings support the model depicted by our prior studies that G incorporation into VLPs may be more tied to the budding activity of NiV F than that of M (18). To further investigate this model and elucidate whether the change from increased (G-only) to decreased (FGM) G incorporation during coexpression of DN-dynamin was driven by the presence of F or M, the budding efficiency of G in both the FG and MG combinations was next assessed with and without cotransfection of DN-dynamin (Fig. 6). Supporting our model, these experiments demonstrated that the addition of DN-dynamin substantially reduces G incorporation in the FG combination and either does not change or supports increased G incorporation in the MG combination (Fig. 6A and B).

### Select mutant cellular factors involved in vesicular trafficking and cytoskeletal organization affect F budding and cell-cell fusion without affecting F processing.

**FIG 7:**
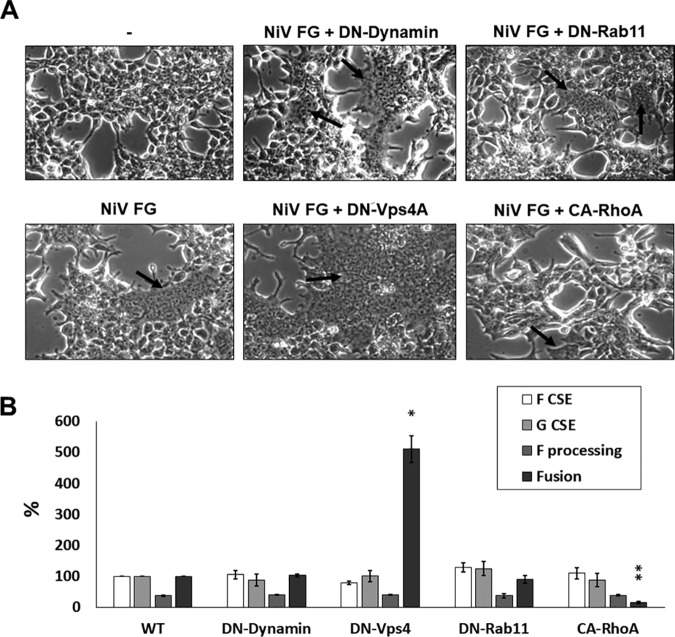
Expression of select mutant factors that inhibit F budding does not affect processing but can modulate fusion. (A) HEK293T cells were transfected with an empty vector or with NiV F and G with or without the listed cellular factors. Light microscope images were taken at ×200 magnification. (B) By counting nuclei in fused syncytia, levels of fusion were quantified, with F and G cotransfection alone set to 100%. Levels of F processing as well as F and G CSE levels were quantified from FGM cotransfections. The results are representative of at least three experiments, with error bars indicating standard error of the mean. One-way Student *t* tests were used to assess significant differences in budding efficiencies for each viral protein compared to FGM coexpression alone (*, *P* < 0.05; **, *P* < 0.01).

While we have so far shown that mutants of several cellular factors involved in vesicular trafficking and in cytoskeletal organization impact NiV budding in the hopes of validating and expanding upon VLP proteomics, the full importance of these major cellular processes in the life cycle of NiV remains unclear. Since proteasomal cleavage of NiV F by endolysosomal cathepsin B or L has been shown to be essential for its fusogenic activity (54–56), we tested whether some of the constructs affected F fusogenicity using a cell-cell fusion assay. While we report that neither DN-dynamin nor DN-Rab11 significantly affected fusion, DN-Vps4a dramatically increased fusion (by 410%, *P < *0.05), while CA-RhoA significantly decreased fusion (to 16%, *P < *0.01) (Fig. 6A). Notably, data from FGM cotransfection experiments (Fig. 5) showed that none of the tested constructs significantly affected F or G CSE nor F processing levels; thus, these parameters were not responsible for the fusogenicity phenotypes observed (Fig. 7B). These findings not only suggest that NiV-F can be internalized or trafficked through the endolysosomal system through dynamin-independent means but also indicate that ESCRT and cytoskeleton machinery are able to modulate both sites of budding and fusion, either directly or indirectly.

## DISCUSSION

While recent studies have helped to clarify how Nipah virus assembly and budding occur, many gaps in our understanding remain. While paramyxovirus matrix proteins are generally accepted as crucial for driving particle formation, incorporating all required viral components as well as supporting the stability and infectivity of produced virions, significantly less is understood concerning how other viral proteins may support particle formation (9, 17). Unlike most paramyxoviruses, NiV F, G, and M have all been reported to autonomously produce VLPs to various degrees upon expression alone, suggesting potential roles as supportive in budding (46). From this finding, it is likely that particles lacking one or more viral components are produced during live infection. While no biological roles have so far been shown for NiV particles lacking F, G, or the internal viral machinery, some such particles may serve a role during infection. One possibility could be the use of FG particles lacking M and/or the vRNP as decoys to attract neutralizing antibodies. Our recent study further suggested a novel role for NiV F as capable of increasing the VLP incorporation of G (18). Moreover, our study implicated the cytoplasmic tail of NiV F as containing several specific motifs important for this budding function (37, 57). The exact function(s) of these motifs remain unclear; however, the presence of a YxxL motif may suggest interaction with the ESCRT-associated protein ALIX and/or other vesicular trafficking machinery (21, 58–60).

In a recent study published by Vera-Velasco et al. (43), VLPs produced from the coexpression of NiV F, G, and M in HEK293 cells were analyzed using liquid chromatography-tandem mass spectrometry (LC-MS/MS)-based proteomics, identifying proteins involved in vesicular trafficking as significantly incorporated (43). While this study gave an exciting first look into cellular proteins and machinery potentially involved in NiV particle formation, the importance of F, G, and M for cellular protein incorporation remained unknown. Additionally, the importance of the incorporated proteins and machinery identified in this study remained unelucidated. Here, we analyzed by proteomics the VLPs produced from either individual expression or various combinations of F, G, and M coexpression, and we validated the most significant processes. Corroborating the findings from the study by Vera-Velasco et al. (43), we report that proteins incorporated into FGM VLPs were most significantly associated with vesicular trafficking (Fig. 2B). We also found that proteins involved in the cytoskeleton showed significant incorporation as a group. Further, we reported that while M-only VLPs incorporated few cellular proteins, all VLP combinations containing F (F alone and FM) largely recapitulated the levels and patterns of cellular factor incorporation observed during FGM expression (Fig. 2). Together, these findings support a model where the matrix protein induces budding based on its ability to form higher-order scaffolds, whereas the fusion protein likely relies on the recruitment of cellular machinery to sites of particle formation and, in some cases, to virions (23, 29, 59). Future proteomics studies focused on posttranslational modifications of viral and cellular factors incorporated into VLPs may further clarify the processes of assembly and budding.

To attempt to validate those VLP proteomic analyses conducted previously (43) and those described here, we targeted several aspects of vesicular trafficking and actin cytoskeletal organization. Supporting our findings that VLPs made with combinations that include F or M tend to incorporate cellular factors associated with vesicle transport (Fig. 2), we report that dominant-negative constructs of dynamin, Eps15, and Rab11 each inhibit F and M budding when coexpressed (Fig. 3). Confirming further reports of M independence from ESCRT machinery (47), we also report that DN-Vps4A significantly reduced F but not M budding. The potential importance of ESCRTs in NiV budding is greatly supported by the identification of multiple ESCRT proteins both in this study and in that by Vera-Velasco et al. (43). Very recently, live NiV infection was shown to be significantly reduced by knockdown of ESCRT machinery, with a new role for the small NiV C protein as a potential ESCRT recruitment factor. As with several retroviruses, numerous viral proteins and motifs (61–64) can be important for ESCRT recruitment, and since neither our study nor that published previously included C, there remains a significant potential for F-driven budding to rely on the direct action of ESCRTs.

Reduced F and M budding during DN-Rab11 cotransfection is further corroborated by previous findings with the closely related Hendra virus (HeV) for which both the F and M proteins have been shown to interact with Rab11-positive recycling endosomes, and their budding is at least partially dependent on Rab11 activity (44). The full function of Rab11 during henipavirus budding is unknown; however, the importance of Rab11 and Rab11-interacting proteins has also been recently highlighted for several more distantly related enveloped viruses, including influenza A virus and respiratory syncytial virus (33, 35).

Our findings from the coexpression of constitutively active and dominant-negative RhoGTPases suggest that the actin cytoskeleton modulates NiV budding and validate the significance of cytoskeletal factor incorporation into NiV VLPs. Further studies are needed to enrich our understanding of potential roles that actin remodeling has in henipavirus particle formation. The potential involvement of the actin cytoskeleton may be in trafficking, assembly, or in the budding process itself (36, 38). Specific RhoGTPases have also been implicated as having roles in different pathways of endocytosis (65–68), which may explain the variation of effects on F, G, and M budding efficiencies (Fig. 4).

To better understand the importance of our budding phenotypes upon vesicular trafficking or cytoskeletal perturbation, FGM VLPs were characterized. We found a very clear phenotype suggesting the dependence of G incorporation on the budding of F regardless of M budding (Fig. 5). Interestingly, these results were supported by our previous findings using a budding-defective F mutant (18). Most striking was the clear shift from DN-dynamin expression increasing G-only budding by almost 7-fold (Fig. 3) to a reduction by nearly the same magnitude when F was expressed (Fig. 5), suggesting that the budding activity of F overrides that of G when they are coexpressed. A potential explanation for this is the observed shift in trafficking profiles of these proteins in a comparison of individual expression and coexpression; however, these changes require further study (58, 69).

Since vesicular trafficking has been previously associated with henipavirus F protein maturation, we also sought to understand how our constructs that most clearly altered budding would affect F processing and fusogenic capacities (56). We found that neither processing nor levels of F or G CSE were altered substantially after coexpression of any of the following constructs tested: DN-dynamin, DN-Vps4A, DN-Rab11, or CA-RhoA. Despite no clear change to processing or CSE, DN-Vps4A and CA-RhoA coexpression led to clear phenotypes of increased and reduced fusion, respectively (Fig. 7).

Somewhat similar reductions in fusion seen with CA-RhoA have been observed with HeV but were not consistent when assessed for parainfluenza 5, another paramyxovirus (70). Thus, the roles of actin cytoskeletal dynamics are not well understood for any aspect of the NiV life cycle. Future study is needed to understand the functions of the cytoskeleton during the life cycles of paramyxoviruses, though these mechanisms are likely to be obscured by differences between cell types and will vary by viral species.

Our observation of increased fusion during DN-Vps4A was completely unexpected and does not seem to be due to increased F processing nor to F or G CSE increases. The cause of this phenotype is unknown but we speculate could be explained by a change in F and G localization within the cell that is more optimal for cellular fusion. Superresolution microscopy may shed further light into the effects of Vps4A in cell-cell fusion.

Here, we have described a VLP proteomics approach of identifying cellular factors and processes potentially involved in NiV assembly and budding. Further, this was accomplished for VLPs produced from several combinations of NiV proteins to clarify the function of several viral proteins in cellular factor incorporation. We validated our VLP proteomics results by demonstrating that perturbation of the most significantly enriched functional groups, vesicular trafficking and the actin cytoskeleton, led to substantial modulation of VLP budding, with the clearest phenotypes being reductions in F-driven budding. The importance of functional F budding during the incorporation of the G protein into VLPs was further demonstrated using these mutant constructs. Together, these findings support a significant involvement of the NiV fusion protein and uncover the involvement of vesicular trafficking and actin cytoskeletal processes in efficient Nipah virus assembly and budding.

## MATERIALS AND METHODS

### Cells, DNA plasmids, and antibodies.

HEK293T cells were obtained from the ATCC and used for all experiments. These cells were grown in Dulbecco’s modified Eagle medium (DMEM; Corning) supplemented with 10% fetal bovine serum (FBS) and 1% penicillin-streptomycin (Gibco) at 37°C with 5% CO_2_. Previously published DNA constructs for NiV proteins, pcDNA3.1-F-AU1, pcDNA3.1-G-HA, and pCMV-3XFLAG-M, were used (18). These constructs were also used to make pCAGGS-NiV-F-AU1 and pCAGGS-NiV-M-3XFLAG. A dominant-negative (DN; S25N) mutant of Rab11 (71) inserted into a pGL vector was generously provided by Ruth Collins (Cornell University). pEGFP constructs containing previously described dominant-negative mutants of dynamin-2 (DN-dynamin; K44A) and eps15 (DN-Eps15; Δ95/295) were used (72–74). pcDNA3.1 constructs containing constitutively active (CA) and dominant-negative RhoA, Rac1, and Cdc42 were used (75). The dominant-negative Vps4A (DN-Vps4A) mutant (76) was synthesized and inserted into a pcDNA3.1 vector (Biomatik). Antibodies used against NiV F were rabbit polyclonal 835term for flow cytometry (77) and mouse monoclonal anti-AU1 for Western blotting (BioLegend). Also used were primary antibodies against G, mouse monoclonal anti-hemagglutinin (HA) for flow cytometry (BioLegend) and rabbit polyclonal anti-HA for Western blotting (Bethyl). Mouse monoclonal M2 anti-FLAG was used to detect NiV M for Western blotting (Sigma-Aldrich). Mouse monoclonal anti-sequestosome-1 antibodies were used to validate VLP proteomics (Abcam, no. ab56416). Goat anti-mouse and anti-rabbit 647 and 488 secondary antibodies (Thermo Fisher Scientific) were used for Western blotting and flow cytometry, as described previously (18).

### NiV VLP production and sample preparation for proteomic analyses.

Five 15-cm^2^ dishes confluent with HEK293T cells were transfected using polyethylenimine (PEI; Polysciences, Inc.) at 1 mg/ml for each of the following combinations of plasmid constructs: pcDNA3.1, F, M, MG, FM, and FGM at an FGM DNA ratio of 24:1:5 F:G:M) (the table in Fig. 1A shows this as well). For each dish, 30 μg of total DNA was transfected at a 4:1 ratio of PEI to DNA, as described previously (78). For these experiments, pCAGGS versions of NiV-F and NiV-M constructs were used for optimal expression. After 48 h, the medium was collected and cleared of cellular debris by centrifugation at 376 × *g* for 10 min, as previously described (18). The resulting supernatant was collected and layered over 20% sucrose in NaCl-Tris-EDTA (NTE) buffer and spun at 110,000 × *g* for 90 min. After this VLP purification spin, VLPs were resuspended with 5% sucrose in NTE buffer and shipped to Pacific Northwest National Laboratories for VLP proteomics analyses, which are described below.

### Sample preparation for VLP proteomics.

Purified viral-like particle samples were lysed and extracted by adding 1.0 ml of 2:1 (vol/vol) chloroform-methanol and vortexing before storing on ice. The samples were then placed in a centrifugal concentrator and dried completely overnight. Proteins were solubilized with the addition of 10 μl of homogenization buffer (8 M urea, 10 mM dithiothreitol, 50 mM Tris [pH 8]), followed by 30 s in a bath sonicator. The lysate was then incubated at 60°C for 30 min to denature and reduce the proteins. The protein solution was quantitatively transferred to a low-retention LC vial (Waters) using 25 μl of 50 mM Tris (pH 8). SNP and AFLP Package for Phylogenetic (SNaPP) analysis was carried out as described previously (79, 80). Briefly, 25 μl of sample was injected onto a 150-μm by 2-cm immobilized enzyme reactor for digestion. The digestion column was syringe packed using Poroszyme immobilized trypsin (Applied Biosystems). Following digestion, peptides were separated using a 100-min gradient on an in-house-packed 50-μm by 75-cm C_18_ analytical column (Phenomenex). The SNaPP system was coupled to a Q Exactive Plus mass spectrometer (Thermo Scientific) operated at a mass resolution of 70 K for MS1 and 17.5 for MS2 collection. Data were collected in data-dependent acquisition (DDA) mode with a top 12 method and a dynamic exclusion window of 30 s. The maximum ion time was increased to 200 ms for MS2 to maximize identifications from low sample loadings.

### Proteomics.

Peptide samples (5 μl) were analyzed by LC-MS/MS using a Waters nano-Acquity M-class dual-pumping ultraperformance liquid chromatography (UPLC) system (Milford, MA) configured for on-line trapping at 5 μl/min for 8 min, followed by gradient elution through a reversed-phase analytical column at 300 nl/min. Columns were packed in-house using 360-μm outside diameter (o.d.) fused silica (Polymicro Technologies, Inc.) and contained Jupiter C_18_ medium (Phenomenex) in 5-μm particle size for the trapping column (100 μm inside diameter [i.d.] by 4 cm long) and 3-μm particle size for the analytical column (75 μm i.d. by 70 cm long). Mobile phases consisted of (MP-A) 0.1% formic acid in water and (MP-B) 0.1% formic acid in acetonitrile with the following gradient profile: 0 min, 1% MP-B; 2 min, 8% MP-B; 20 min, 12% MP-B; 75 min, 30% MP-B; 97 min, 45% MP-B; 100 min, 95% MP-B; and 110 min, 95% MP-B.

MS analysis was performed using a Q-Exactive high-fidelity (HF) mass spectrometer (Thermo Scientific, San Jose, CA). Electrospray emitters were prepared in-house using 150-μm o.d. by 20-μm i.d. chemically etched fused silica (89), subsequently attached to the column using a metal union, and coupled to the mass spectrometer via a custom-built nanospray source. Electrospray voltage (2.2 kV) was applied at the metal union providing ions to the heated (325°C) ion transfer tube entrance of the MS. Data collection was started 20 min after the gradient began and continued for a total acquisition time of 100 min. Precursor mass spectra (MS) were acquired from *m/z* 400 to 2,000 at a resolution of 60 K (automatic gain control [AGC] target, 3e6; max IT, 20 ms), followed by data-dependent MS/MS spectra of the top 12 most abundant ions from the precursor spectrum with an isolation window of *m/z* 2.0 and at a resolution of 15 K (AGC target 1e5; max IT, 200 ms) using a normalized collision energy of 30 and a 45-s exclusion time.

### VLP proteomics analyses.

Modern mass spectrometers can observe very low-abundance contaminants in low-complexity samples, such as VLPs. Further, the experimental enrichment of very small particles with subsequent sample preparation by SNaPP analysis results in an approximation of loading protein quantity. Hence, samples are batched as sets and then randomized within sets to allow for the interpretation of potential carryover from abundant proteins from one VLP composition measurement to another. Relative quantification was performed via a spectral counting approach where the number of tandem mass spectra identified as matching a protein is used as a proxy for abundance that can be compared across data sets.

Further, the identification of peptides corresponding to the three NiV proteins, F, G, and M, was used to evaluate the quality of measurements. LC-MS/MS analyses considered for further analysis were included or excluded based on expected viral protein compositions. Specifically, we set the requirement that a replicate would only be included in the next stages of analysis if it contained no more than 2 observed peptides corresponding to unexpected viral protein carryover (e.g., M peptides in an F sample replicate). Similarly, we also set the requirement that each included replicate would require at least two observed peptides for each expected viral protein. The potential limitations of this were that the viral proteins did have different observation rates due to different lengths and expression levels; however, levels of peptides for viral proteins within samples they were expected were almost always much greater than in samples in which they were unexpected, regardless of the viral protein.

To identify host-derived proteins considered to be incorporated into VLPs of each combination, an approach based on averaged observed peptides was used. All proteins considered incorporated in any type of VLP were not allowed to have any more than an average observed peptide count of 2 in the control samples (VLPs isolated from cells transfected with an empty vector) and were required to have at least an average of 4.33 observed peptides in the VLP type of interest. By setting a low maximum threshold for observed peptides in the empty vector control, we sought to remove cellular factors highly enriched in background exosomes and microvesicles. An average observed peptide count of 4.33 in VLPs was set as the threshold, as this corresponded to the lowest level of average peptide incorporation for any expected viral protein in any of the VLP combination types.

After the incorporated host-protein protein lists were determined for each VLP combination, comparisons were done using InteractiVenn (81). Further, protein-protein interaction mapping was completed using the STRING and Cytoscape softwares (82, 83). Gene ontological designations used for Table 1 and the protein interaction map were conducted using GOTermFinder (84).

### VLP budding assay.

HEK293T cells were transfected at between 65% and 85% confluence using 1 mg/ml PEI. After 24 h, medium was collected and precleared by centrifugation as described above, while the transfected cells were harvested with 10 mM EDTA (from 0.5 M stock; VWR) in Dulbecco’s phosphate-buffered saline (DPBS; Corning) and split into two groups, the cell lysate and cell surface expression. For both groups, cells were pelleted by centrifugation at 376 × *g* for 10 min and the supernatant removed. For the cell lysate fraction, 1× radioimmunoprecipitation assay (RIPA) buffer (Sigma-Aldrich) was prepared in tissue culture-grade water (Corning) with the cOmplete Ultra protease inhibitor (Roche) and added to the pellet prior to 30 min of vortexing and rotating at 4°C. The lysate supernatant was collected and prepared for 10% SDS-PAGE (Protogel), transferred to an activated polyvinylidene difluoride (PVDF) membrane (Thermo Fisher Scientific), and blotted with antibodies against AU1, HA, and FLAG tags, as described previously (18). For the group to be assessed for cell surface expression, the pellet was resuspended with FACS buffer (1% FBS in DPBS) and stained with rabbit polyclonal 835term (for F) and mouse monoclonal anti-HA (for G) for 1 h prior to three washes with FACS buffer, secondary antibody staining for 30 min, two final FACS washes, and analysis with a Millipore Guava easyCyte flow cytometer 8HT; 10,000 or more cells were analyzed per sample, as described previously (18).

### Transmission electron microscopy sample preparation and imaging.

One 15-cm dish of HEK293T cells was transfected using PEI, as discussed above, for each combination of F, G, and M. VLPs were harvested and negatively stained with 1% uranyl acetate and visualized using a Tecnai T12 Spirit microscope (FEI) at 120 kV located in the Cornell University Center for Materials Research.

### Cell-cell fusion assay.

HEK293T cells were transfected at 80 to 95% confluence with NiV F, G, and either pcDNA3.1 or one of the mutant cellular factors as described at a DNA:PEI ratio of 1:3 with a total transfection of 2.1 μg of DNA (F:G:pcDNA or cell factor ratio of 3:1:3). After 12 h, the cells were fixed in 1% paraformaldehyde at 37°C for 1 h and imaged on a Leica bright-field microscope. Five fields of view at ×200 magnification were used to quantify levels of cell-cell fusion, where each nucleus associated with a syncytium of four or more nuclei was counted. For each transfected sample, the fields of view were averaged, reduced by background syncytial levels (empty vector control), and normalized to the positive control (set at 100%) (85–88).
